# Cytotoxic and inflammatory potential of a phospholipase A_2_ from *Bothrops jararaca* snake venom

**DOI:** 10.1186/s40409-018-0170-y

**Published:** 2018-11-23

**Authors:** Rafhaella C. A. Cedro, Danilo L. Menaldo, Tássia R. Costa, Karina F. Zoccal, Marco A. Sartim, Norival A. Santos-Filho, Lúcia H. Faccioli, Suely V. Sampaio

**Affiliations:** 1 Departamento de Análises Clínicas, Toxicológicas e Bromatológicas, Faculdade de Ciências Farmacêuticas de Ribeirão Preto, Universidade de São Paulo (FCFRP-USP), Avenida do Café, s/n, B. Monte Alegre, Ribeirão Preto, SP 14040-903 Brazil; 2 Campus Experimental de Registro, Universidade Estadual Paulista (UNESP), Registro, SP Brazil

**Keywords:** Snake venom, *Bothrops jararaca*, Phospholipase A_2_, Inflammation, Cytotoxicity

## Abstract

**Background:**

Snake venom phospholipases A_2_ (PLA_2_s) have been reported to induce myotoxic, neurotoxic, hemolytic, edematogenic, cytotoxic and proinflammatory effects. This work aimed at the isolation and functional characterization of a PLA_2_ isolated from *Bothrops jararaca* venom, named BJ-PLA_2_-I.

**Methods and Results:**

For its purification, three consecutive chromatographic steps were used (Sephacryl S-200, Source 15Q and Mono Q 5/50 GL). BJ-PLA_2_-I showed acidic characteristics, with pI~ 4.4 and molecular mass of 14.2 kDa. Sequencing resulted in 60 amino acid residues that showed high similarity to other *Bothrops* PLA_2_s, including 100% identity with BJ-PLA_2_, an Asp49 PLA_2_ previously isolated from *B. jararaca* venom. Being an Asp49 PLA_2_, BJ-PLA_2_-I showed high catalytic activity, and also inhibitory effects on the ADP-induced platelet aggregation. Its inflammatory characterization showed that BJ-PLA_2_-I was able to promote leukocyte migration in mice at different concentrations (5, 10 and 20 μg/mL) and also at different response periods (2, 4 and 24 h), mainly by stimulating neutrophil infiltration. Furthermore, increased levels of total proteins, IL-6, IL-1β and PGE_2_ were observed in the inflammatory exudate induced by BJ-PLA_2_-I, while nitric oxide, TNF-α, IL-10 and LTB_4_ levels were not significantly altered. This toxin was also evaluated for its cytotoxic potential on normal (PBMC) and tumor cell lines (HL-60 and HepG2). Overall, BJ-PLA_2_-I (2.5–160 μg/mL) promoted low cytotoxicity, with cell viabilities mostly varying between 70 and 80% and significant values obtained for HL-60 and PBMC only at the highest concentrations of the toxin evaluated.

**Conclusions:**

BJ-PLA_2_-I was characterized as an acidic Asp49 PLA_2_ that induces acute local inflammation and low cytotoxicity. These results should contribute to elucidate the action mechanisms of snake venom PLA_2_s.

## Background

Phospholipases are lipolytic enzymes classified as A_1_, A_2_, B, C or D, according to the position where they induce lipid hydrolysis [1]. Phospholipases A_2_ (PLA_2_s) catalyze the hydrolysis of fatty acids at the sn-2 position of the phospholipid membranes, and release lysophospholipids and free fatty acids, especially polyunsaturated ones, such as arachidonic acid. Based on their structure, catalytic mechanisms, localization and evolutionary interactions, the PLA_2_s can be divided into 6 major families and 15 subgroups, with snake venom PLA_2_s being classified as secreted PLA_2_s (sPLA_2_) from groups I (Elapidae and Hydrophiidae snakes) or II (Viperidae and Crotalidae snakes) [2, 3].

In general, snake venom PLA_2_s are acidic or basic enzymes with molecular masses ranging from 13 to 15 kDa, and structure consisting of about 120 amino acid residues stabilized by 7 disulfide bonds, making them very stable molecules. They present a highly conserved catalytic site formed by the amino acid residues His48, Asp49, Tyr52 and Asp99. Aspartic acid residue at position 49 coordinates the hydrolysis reaction of phospholipids together with the residues of the Ca^2+^ binding loop, with this ion being an essential cofactor in the catalytic activity of PLA_2_s. Also commonly reported in the literature is the existence of PLA_2_ homologues with a mutation at position 49 that exchanges the aspartic acid residue for a lysine. These toxins are called Lys49 PLA_2_-like molecules, and this amino acid substitution leads to partial or total loss of their catalytic activity [4, 5]. Thus, Asp49 PLA_2_s present high catalytic activity, while Lys49 PLA_2_-like molecules do not, but can still induce several biological effects, such as myonecrosis, inflammation and cytotoxicity [4, 6–8].

PLA_2_s are usually among the most abundant components of snake venoms, being responsible for various toxic and pharmacological effects, by mechanisms not yet fully understood [9]. During envenomations, they assist in the prey digestion, and have also been described to induce myotoxic, neurotoxic, cytotoxic, hemolytic, edematogenic, hypotensive, anticoagulant, platelet aggregation inhibition/activation, bactericidal and proinflammatory effects [10].

Considering that snake venom PLA_2_s can act directly on phospholipid membranes, they should be able to promote alterations in lipid biosynthesis and dysregulation of lipogenesis that could have great impact on the metabolism of tumor cells and also on the formation of lipid mediators derived from arachidonic acid, which perform essential roles in inflammation. Hence, such PLA_2_s might serve as useful tools to elucidate the mechanisms involved in cancer/inflammation and as possible molecular models for new antitumor/anti-inflammatory drugs [11–14]. In fact, different PLA_2_s have been studied for their proinflammatory, antitumor and antiangiogenic properties, among them acidic and basic PLA_2_s, as well as synthetic peptides derived from Lys49 PLA_2_ homologues [11, 15].

Therefore, research on PLA_2_s has attained paramount importance, not only to better understand the role of these toxins in envenomations, but also to discover molecular and biotechnological tools for the formulation of new drugs to combat inflammatory diseases and cancer. Thus, this study aims to evaluate the cytotoxic and inflammatory effects of an acidic phospholipase A_2_ isolated from *Bothrops jararaca* snake venom.

## Methods

### Venom and other materials

*Bothrops jararaca* venom was extracted and processed in the Laboratory of Herpetology of the Butantan Institute (São Paulo, Brazil), which then kindly donated it for the development of the present study. The chromatographic resins and reagents for the biochemical and enzymatic assays were obtained from GE Healthcare, Merck, Thermo Scientific or Sigma-Aldrich. Other materials and equipment used were described throughout the methodology. Unless otherwise specified, reagents were of analytical grade.

### Animals

Male BALB/c mice (20–25 g, 6–8 weeks old) used in the inflammatory experiments were bred and provided by the animal facilities of the University of São Paulo, campus of Ribeirão Preto (São Paulo, Brazil).

### Human blood

The human plasma used in the platelet aggregation experiments and the peripheral blood mononuclear cells (PBMC) for cytotoxicity assays were obtained from blood donated by healthy volunteers aged 20–40 years, from both sexes, and who had not been using any medication for 10 days prior to the collection.

### Toxin isolation

To isolate the PLA_2_ from *B. jararaca* venom, we used three consecutive chromatographic steps: (i) Sephacryl S-200 molecular exclusion chromatography, (ii) Source 15Q anion exchange chromatography and (iii) Mono Q 5/50 GL anion exchange chromatography.

Firstly, *B. jararaca* crude venom (200 mg) was suspended in 2 mL of 50 mM ammonium bicarbonate buffer (Ambic) pH 8.0, followed by centrifugation at 10,000×g for 10 min at room temperature. Next, the supernatant was applied to a Sephacryl S-200 column (127 × 3.5 cm), previously equilibrated and eluted with the same buffer at room temperature, collecting fractions of 3 mL/tube at a flow rate of 15 mL/h. All fractions were monitored in a Beckman DU® 640 spectrophotometer, using a wavelength of 280 nm, and pools were separated based on the chromatographic profile. SDS-PAGE and phospholipase activity were employed to define the pool of interest (identified as fraction F), which was then submitted to the next chromatographic step.

For the second step, the lyophilized fraction F (~ 50 mg) was suspended in 1 mL of 20 mM Tris-HCl buffer, pH 8.0, and centrifuged at 10,000×g for 10 min. The clear supernatant was applied to a Source 15Q column (11.5 × 2.6 cm), previously equilibrated at room temperature with 20 mM Tris-HCl buffer, pH 8.0. Fractions were eluted using an AKTA FPLC system (GE Healthcare) and a linear gradient of NaCl (from 0 to 1 M), collecting fractions of 3 mL/tube at a flow rate of 1 mL/min. Absorbance was monitored at 280 nm and, once again, SDS-PAGE and phospholipase activity were utilized to determine the pool of interest (identified as fraction S.10).

For the third chromatographic step, the lyophilized fraction S.10 (~ 1.2 mg) was suspended in 550 μL of 50 mM Ambic buffer, pH 8.0, and centrifuged at 10,000×g for 10 min. The supernatant was then applied to a Mono Q 5/50 GL column (5 × 0.5 cm), previously equilibrated at room temperature with 50 mM Ambic buffer, pH 8.0. Fractions were eluted using an AKTA FPLC system (GE Healthcare) and a linear gradient of NaCl (from 0 to 1 M), collected at 0.5 mL/tube at a flow rate of 1 mL/min, and monitored for absorbance at 280 nm.

The major peak from the latter chromatographic step was denominated BJ-PLA_2_-I and was then evaluated for its purity by reversed-phase chromatography. For that, lyophilized BJ-PLA_2_-I (~ 200 μg) was dissolved in solution A (0.1% trifluoroacetic acid - TFA) and centrifuged at 10,000×g for 10 min; next the resulting supernatant was applied to a CLC-ODS C18 reversed-phase column (25 × 0.46 cm) using a HPLC system (Shimadzu Biotech). The elution was performed at a flow rate of 1 mL/min with a linear concentration gradient of solutions A and B (70% acetonitrile and 0.1% TFA), as follows: 100% solution A (15 min), 0–100% solution B (50 min), 100% solution B (10 min). Absorbance of fractions was monitored at 280 nm.

### Polyacrylamide gel electrophoresis in the presence of sodium dodecyl sulfate (SDS-PAGE)

SDS-PAGE of chromatographic fractions and the purified toxin was performed according to Laemmli [16], using 12% polyacrylamide gels and reducing or non-reducing conditions (presence or absence of β-mercaptoethanol, respectively). The molecular mass standard used was from Thermo Scientific (ref #26610) and ranged from 14.4 to 116 kDa.

### Protein quantification

Protein was quantified using the Pierce BCA Protein Assay Kit (Thermo Scientific, ref. #23225), following the manufacturer’s instructions.

### Molecular mass determination

Molecular mass analyses were performed using an AXIMA Performance MALDI-TOF/TOF mass spectrometer (Shimadzu Biotech), acquiring mass spectra ranging from 3000 to 80,000 m/z in positive linear mode.

### Isoelectric focusing

Isoelectric focusing separations were performed as described by Arantes et al. [17], using a 7% polyacrylamide gel containing carrier ampholytes with pH ranging from 3 to 10 (Pharmalyte, Sigma-Aldrich).

### Circular dichroism spectrometry

Spectra were obtained at wavelengths between 180 and 260 nm with a JASCO J-815 circular dichroism (CD) spectrophotometer using a nitrogen flush in 1 mm path length quartz cuvettes at room temperature. To investigate the conformational changes, spectra were recorded in 0.01 M Tris-HCl pH 7.5 at a protein concentration of 0.5 mg/mL. CD spectra were typically recorded as an average of 10 scans, which were obtained in millidegrees.

### Amino acid sequence determination

The partial amino acid sequence of BJ-PLA_2_-I was determined by a combination of Edman degradation and MALDI-TOF mass spectrometry techniques. N-terminal sequencing was performed in an automatic protein sequencer (PPSQ-33A system, Shimadzu Biotech), using ~ 200 pmol of the toxin. For the mass spectrometry sequencing, the toxin was first subjected to enzymatic digestion with trypsin (Promega Corp.) for 24 h at 37 °C. After that period, tryptic peptides from the reaction were purified on ZipTip columns (POROS R2, Perseptive Biosystems) and then resuspended in a matrix containing α-cyano-4-hydroxycinnamic acid (10 mg/ml); analyses were performed in a MALDI-TOF/TOF mass spectrometer (4800-Plus, Applied Biosystems).

The results generated were compared to sequences deposited in the NCBI and Swiss-Prot databases using the sites BLAST (http://blast.ncbi.nlm.nih.gov/) and MASCOT (http://www.matrixscience.com/search_form_select.html). The partial sequence of BJ-PLA_2_-I was then aligned to sequences of other PLA_2_s deposited in the NCBI database using the program ClustalX v.2.0.11 (http://www.clustal.org/).

### Molecular modeling

The crystallographic model of BthA-I-PLA_2_ from *Bothrops jararacussu* venom (PDB id: 1ZLB) [18] was chosen as the best model for the construction of the theoretical structural model of BJ-PLA_2_-I (100% probability, E-value: 1.3 e^− 37^, according to HHpred), using the MODELLER program (https://toolkit.tuebingen.mpg.de/#/tools/modeller) [19, 20]. The analyses of the obtained models were carried out by three different methodologies, using the programs PROCHECK (https://swissmodel.expasy.org/), VERIFY 3D (http://servicesn.mbi.ucla.edu/Verify3D/) and WHAT IF (http://swift.cmbi.ru.nl/servers/html/index.html). All figures resulting from these studies were constructed by the program PYMOL v1.7.4.4.

### Phospholipase activity

The phospholipase activity of the chromatographic fractions and BJ-PLA_2_-I was evaluated on egg yolk-agar plates, following the methodology described by Gutiérrez et al. [21], with modifications by Menaldo et al. [22]. Assessed samples included a negative control of phosphate buffered saline (PBS), a positive control of *B. jararaca* venom (15 μg) and different quantities of BJ-PLA_2_-I (0.08–2.5 μg), all diluted in PBS. After an overnight incubation of the samples on plates at 37 °C, the phospholipase activity was expressed as the size (in cm) of translucent halos formed by each sample.

### Effects on platelets

Platelet aggregation inhibitory assays were based on the turbidimetric method of Born [23], using platelet-rich plasma (PRP) and adenosine diphosphate (ADP) as agonist. PRP was obtained from blood collected by venipuncture using 3.8% sodium citrate (9:1, *v*/v) as anticoagulant and then centrifuged at 200×g and room temperature for 10 min. After collecting the PRP, the same blood tubes were centrifuged again, this time at 2000×g for 15 min, to obtain platelet-poor plasma (PPP). Plasma platelet counts were performed in a Neubauer chamber, obtaining an approximate value of 2.5 × 10^5^ platelets/mL.

The assays were performed using a platelet aggregometer (Chrono-log Corporation, model 490 2D) and the software AggroLink. Initially, PRP was incubated at 37 °C for 5 min, and then 5 μM ADP was added to determine the percentage of platelet aggregation. Next, PRP was incubated at 37 °C for 5 min, and then for another 5 min with BJ-PLA_2_-I (20.5 μg/mL). After this period, 5 μM ADP was added to the tube to induce platelet aggregation, and the reaction was assayed for additional 10 min. Results were expressed as percentages of platelet aggregation.

### Inflammatory effects

#### Leukocyte recruitment

This evaluation was performed essentially as described by Menaldo et al. [24]. Initially, BALB/c mice (5 animals/group) were injected intraperitoneally (i.p.) with sterile PBS (negative control) or different concentrations of BJ-PLA_2_-I (5, 10 and 20 μg/mL) and animals were euthanized after 4 h by instillation of CO_2_. Then, their peritoneal cavities were washed with cold PBS, and exudates were used to perform the total and differential leukocyte counts.

Subsequently, the same protocol was repeated using a single concentration of BJ-PLA_2_-I (10 μg/mL, equivalent to a dose of 0.12 mg/kg) and different stimulation periods (2, 4 and 24 h). After counting, peritoneal exudates were centrifuged at 400×g for 10 min at 10 °C and the cell-free supernatants were used for the quantification of total proteins, soluble mediators and nitric oxide (NO).

#### Quantification of total proteins

The total protein levels in the peritoneal supernatants from mice injected with BJ-PLA_2_-I or PBS were quantified using Bradford reagent (Sigma-Aldrich), according to the manufacturer’s instructions.

#### Quantification of mediators

The concentrations of cytokines (TNF-α, IL-6, IL-1β and IL-10) and eicosanoids (PGE_2_ and LTB_4_) in the cell-free peritoneal fluid from mice injected with BJ-PLA_2_-I or PBS were quantified by ELISA kits, according to the manufacturer’s instructions (R&D Systems or Cayman Chemical).

#### Quantification of nitric oxide

NO production was determined by the quantification of nitrite (NO_2_^−^) in the peritoneal exudates of mice using a colorimetric assay based on the Griess reaction [25].

#### Cytotoxic effects

##### Cell cultures

Human normal or tumor cells were used in the cytotoxicity experiments, i.e. PBMC (peripheral blood mononuclear cells), HL-60 (human promyelocytic leukemia) and HepG2 (human liver carcinoma). PBMC was obtained from human blood collected in heparin tubes (BD vacutainer ref. #367874) and separated by Histopaque-1077 (Sigma-Aldrich ref. #10771), according to the manufacturer’s instructions. The tumor cell lines HL-60 (CCL-240) and HepG2 (HB-8065) were obtained from ATCC (American Type Culture Collection, Rockville, MD, USA). Before treatments, PBMC and HL-60 cells were cultured for 24 h at 37 °C in RPMI-1640 medium, while HepG2 was cultured in DMEM (Dulbecco’s Modified Eagle Medium), according to Costa et al. [26].

##### Cytotoxicity assays

Cell viability was assessed by the MTT method [27]. PBMC, HL-60 and HepG2 cells were treated with different concentrations of BJ-PLA_2_-I (2.5–160 μg/mL) for 24 h at 37 °C in a CO_2_ incubator. MTT solution was added to the cultures (500 μg/mL, final concentration) 3 h before the end of treatments, and the reaction was stopped by the addition of DMSO (100 μL) to the cell cultures. Cells treated only with sterile PBS were used as negative controls whereas cells treated with cisplatin (Incel, Darrow®) at 250 μg/mL (final concentration) as positive controls. Results were expressed as percentage of cell viability in comparison to the negative controls.

### Statistical analysis

Statistical analysis of the results was performed by the software GraphPad Prism 5, using the Student’s *t* test or one-way ANOVA method with Tukey’s post-test, comparing all treatments to the negative controls and considering values of *p* < 0.05 as significant.

## Results

### Isolation of BJ-PLA_2_-I

**Fig. 1:**
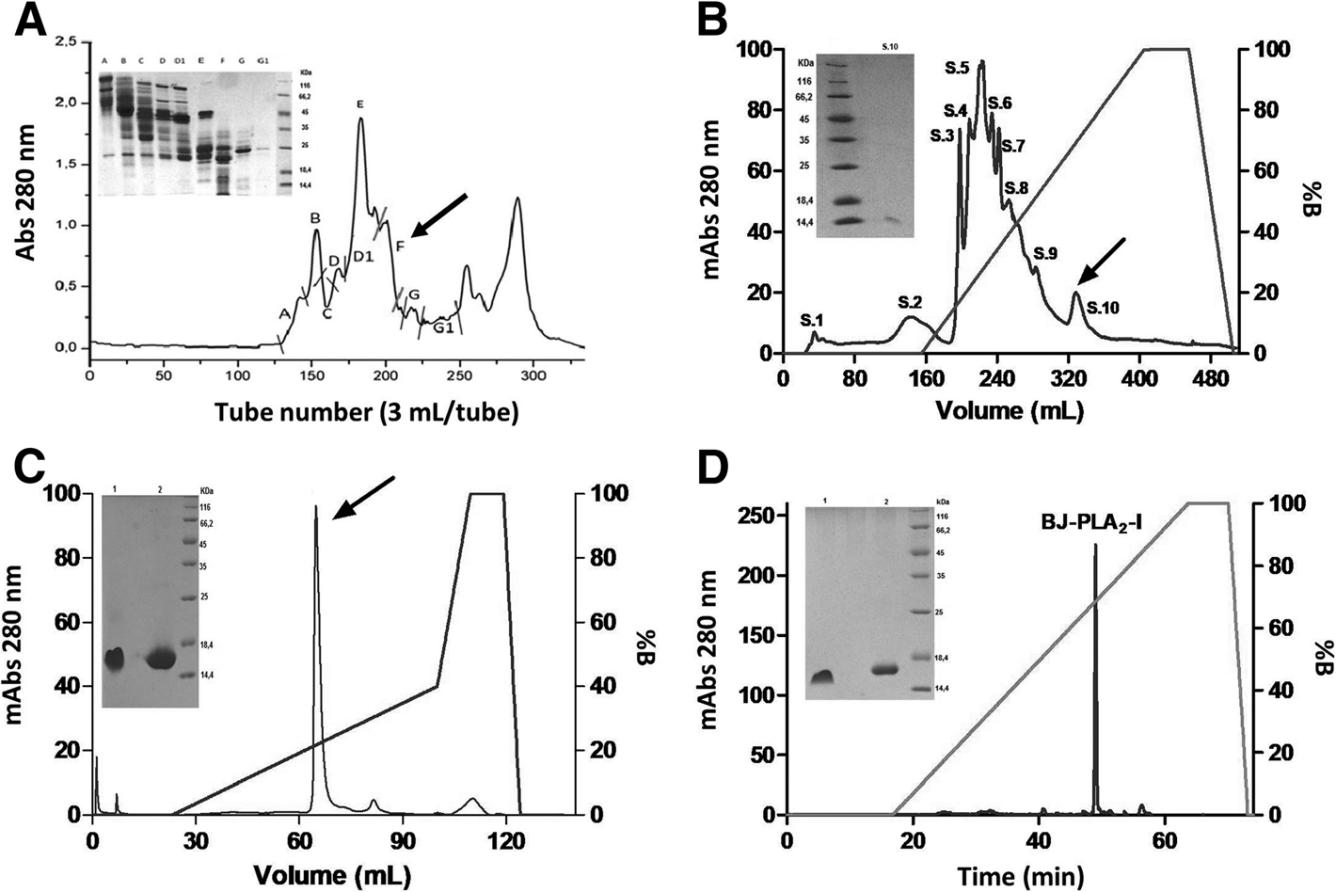
Isolation of BJ-PLA_2_-I from *B. jararaca* venom. **a** Chromatographic profile of *Bothrops jararaca* venom (200 mg) on Sephacryl S-200 column: Elution was carried out with 50 mM Ambic, pH 8.0. Inset, 12% SDS-PAGE of the fractions from A to G1 in non-reducing conditions. **b** Chromatographic profile of fraction F (~ 50 mg) on a Source 15Q column: Elution was carried out with 20 mM Tris-HCl, pH 8.0, and a linear concentration gradient of NaCl (from 0 to 1 M). Inset, 12% SDS-PAGE of the fraction of interest (arrow) in non-reducing conditions. **c** Chromatographic profile of fraction S.10 (~ 1.2 mg) on a Mono Q 5/50 GL column: Elution was carried out with 50 mM Ambic, pH 8.0, and a linear concentration gradient of NaCl (from 0 to 1 M). Inset, 12% SDS-PAGE of the fraction of interest (arrow) in non-reducing (1) and reducing (2) conditions. **d** Chromatographic profile of BJ-PLA_2_-I (~ 200 μg) on a C18 reversed-phase column using a HPLC system: The column was previously equilibrated with solution A (0.1% TFA), and elution was performed at flow rate of 1 mL/min with a linear concentration gradient of solution B (70% acetonitrile and 0.1% TFA). Inset, 12% SDS-PAGE of BJ-PLA_2_-I in non-reducing (1) or reducing (2) conditions

*B. jararaca* venom fractionation was initiated with a molecular exclusion chromatography on Sephacryl S-200, which resulted in several protein fractions that were named A to G1 (Fig. 1a). Fraction F was selected for the next chromatographic step considering its positive phospholipase activity and its protein profile on SDS-PAGE. The following chromatography on a Source 15Q anion exchange column resulted in fractions denominated S.1 to S.10 (Fig. 1b), with fraction S.10 being chosen according to the above mentioned parameters. After the third chromatographic step on a Mono Q anion exchange column (Fig. 1c), the toxin of interest, named BJ-PLA_2_-I, was identified as the major fraction that showed molecular mass around 14 kDa and phospholipase activity. Thus, BJ-PLA_2_-I was successfully isolated from *B. jararaca* venom after these three chromatographic steps, with high purity levels shown by reversed-phase HPLC (Fig. 1d), but very low recovery (~ 0.2%) (Table 1).

**Table 1:** Recovery rates of BJ-PLA_2_-I purification process

	Total protein (mg)^a^	Recovery (%)
*B. jararaca* venom	144.2	100
Fraction F (Sephacryl S-200)	15.2	10.5
Fraction S.10 (Source 15Q)	1.2	0.8
BJ-PLA_2_-I (Mono Q 5/50 GL)	0.28	0.2

^a^Protein concentration determined by the BCA method

### Biochemical, functional and structural characterization

**Fig. 2:**
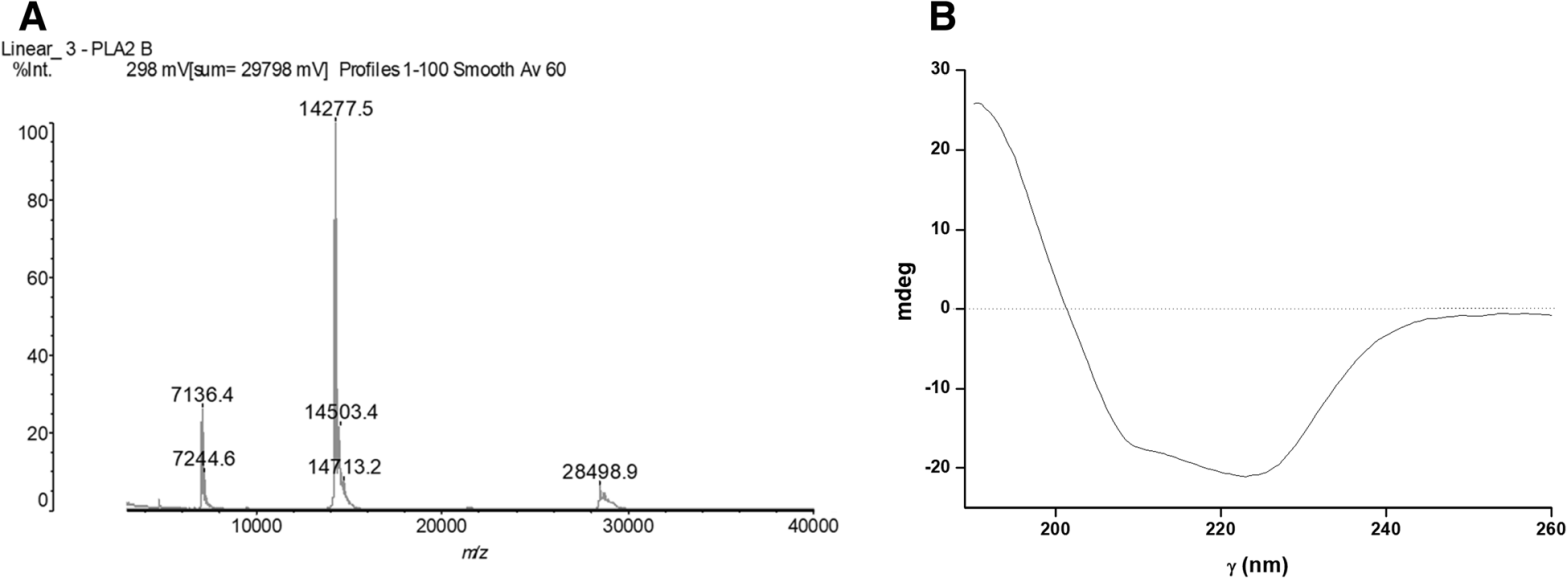
**a** Molecular mass of BJ-PLA_2_-I determined by MALDI-TOF mass spectrometry (MM~ 14.2 kDa). **b** Circular dichroism spectrum of BJ-PLA_2_-I

Once BJ-PLA_2_-I was purified, we performed different assays in order to characterize the toxin. Its molecular mass determined by MALDI-TOF mass spectrometry was of 14,276 Da (Fig. 2a), while its pI was approximately 4.4 as determined by isoelectric focusing (data not shown), thus showing an acidic character.

**Fig. 3:**
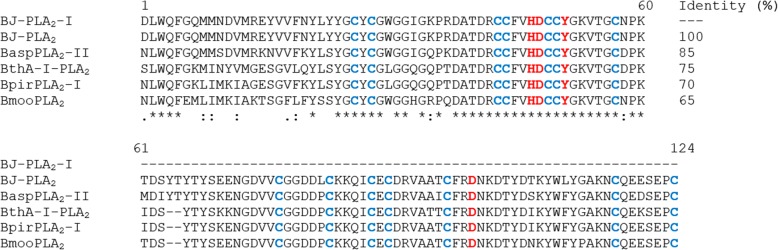
Multiple alignment of the partial amino acid sequence of BJ-PLA_2_-I with sequences of other phospholipases A_2_ from *Bothrops* venoms. Cysteine residues responsible for the formation of disulfide bonds are highlighted in blue and amino acid residues belonging to the catalytic site are in red. The toxins selected for alignment were: BJ-PLA_2_ from *B. jararaca* (gi: 3914258) [28], BaspPLA_2_-II from *B. asper* (gi: 292630844) [58], BthA-I-PLA_2_ from *B. jararacussu* (gi: 82211983) [18], BpirPLA_2_-I from *B. pirajai* (gi: 357580469) [32] and BmooPLA_2_ from *B. moojeni* (gi: 403399514) [33]. (*) indicates positions with fully conserved amino acid residues; (:) indicates conservation of amino acid groups with high score; (.) indicates conservation of amino acid groups with a lower score

**Fig. 4:**
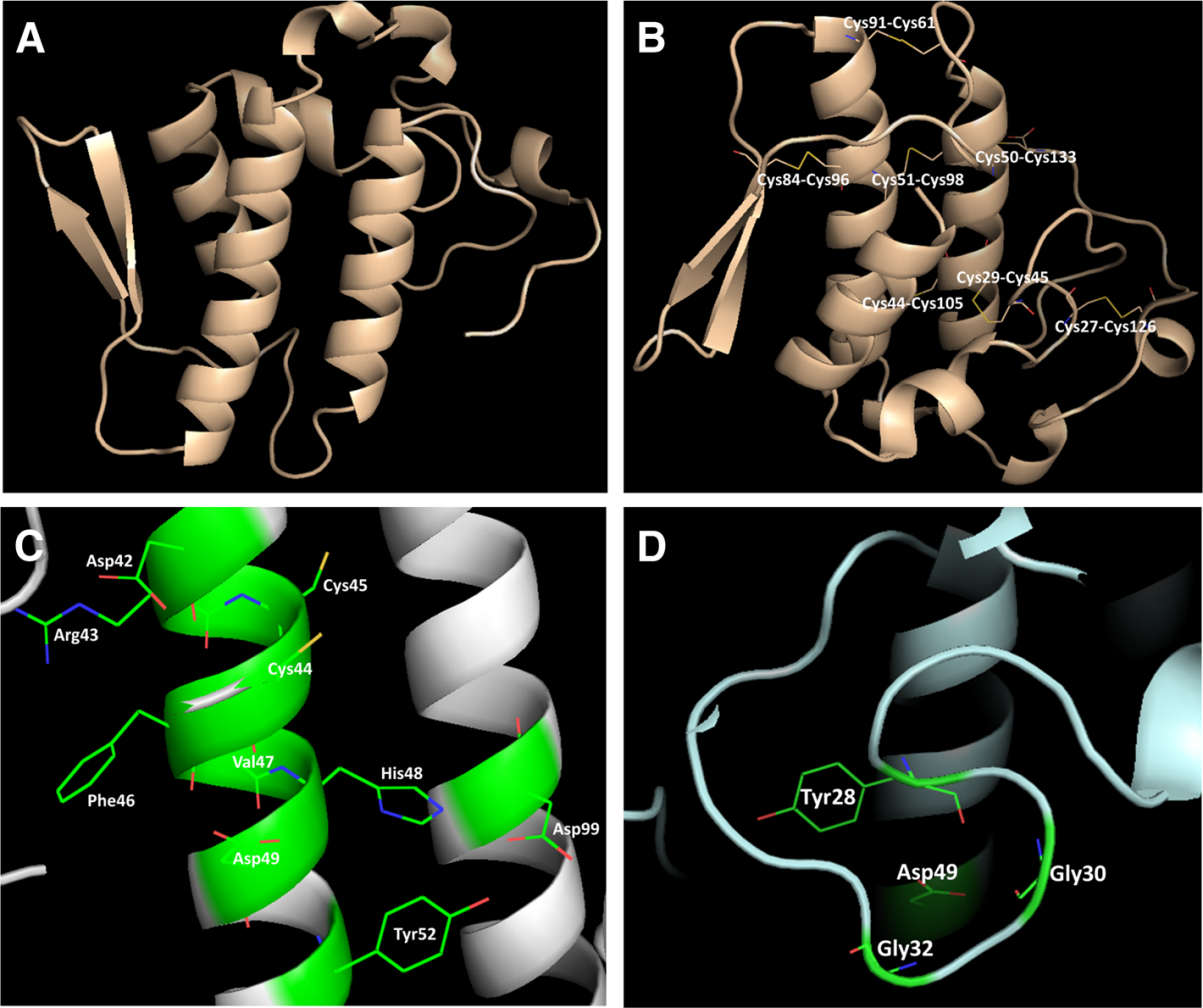
Molecular modeling of BJ-PLA_2_-I. The theoretical structural model of BJ-PLA_2_-I (**a**) was generated by the program MODELLER, using the crystal structure of BthA-I-PLA_2_ from *B. jararacussu* venom (PDB id: 1ZLB) [18] as model. Highlighted are the seven intrachain disulfide bridges formed (**b**), the amino acid residues from the conserved catalytic site (**c**) and the Ca^2+^ binding loop (**d**)

The secondary structure content of the PLA_2_ was analyzed by CD spectroscopy, showing characteristic curves of helical proteins with well-defined peaks at 208 and 222 nm (Fig. 2b). Its partial amino acid sequence was achieved combining Edman degradation and MALDI-TOF mass spectrometry techniques, resulting in 60 amino acid residues from its N-terminal, including 7 cysteine residues and 3 residues belonging to its catalytic site (His48, Asp49 and Tyr52) (Fig. 3). When this sequence was aligned with sequences from other *Bothrops* PLA_2_s, the identity varied from 65 to up to 100% (Fig. 3). Molecular modeling of BJ-PLA_2_-I (Fig. 4a) was made based on the crystal structure of the acidic BthA-I-PLA_2_ from *B. jararacussu* venom, and was useful for illustrating the seven intrachain disulfide bridges formed (Cys27- Cys126, Cys29-Cys45, Cys44-Cys105, Cys50-Cys133, Cys51-Cys98, Cys61-Cys91, and Cys84-Cys96) (Fig. 4b), the conserved catalytic site (D42XCCXXHD49; Tyr52; Asp99) (Fig. 4c) and the amino acid side chains essential for the Ca^2+^ binding (Tyr28; Gly30; Gly32; Asp49) (Fig. 4d).

**Fig. 5:**
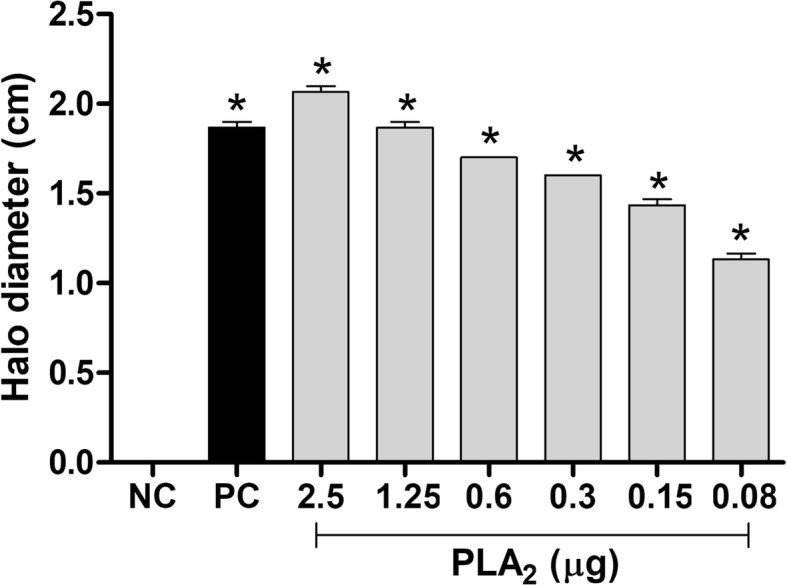
Phospholipase activity of BJ-PLA_2_-I. The assay was evaluated on egg yolk-agar plates incubated overnight at 37 °C with PBS (NC = negative control), *B. jararaca* venom (15 μg) (PC = positive control) or BJ-PLA_2_-I (0.08–2.5 μg). The phospholipase activity is relative to the size (in cm) of translucent halos formed by each sample. Results were expressed as means ± SD (*n* = 2) of two independent experiments. *p < 0.05 compared to the negative control (NC)

**Fig. 6:**
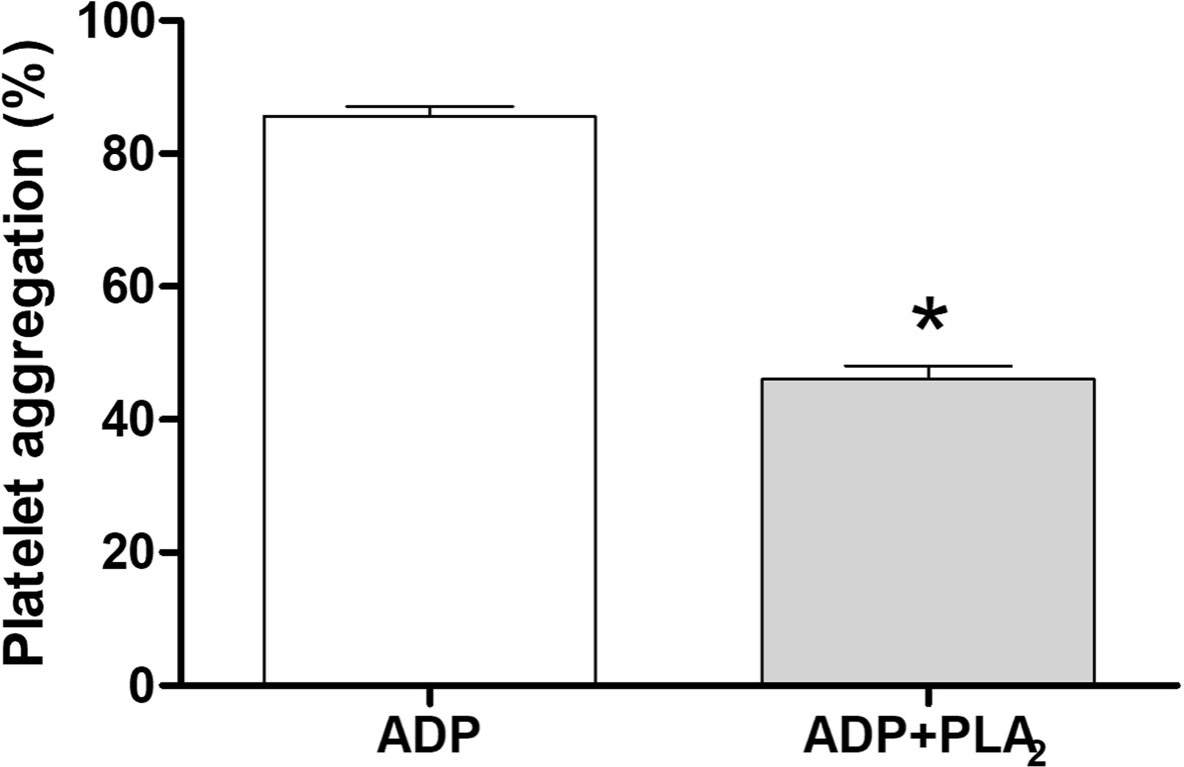
Inhibition of platelet aggregation by BJ-PLA_2_-I. Initially, platelet-rich plasma (PRP) was incubated at 37 °C with ADP (5 μM) for 10 min to determine the percentage of platelet aggregation. Next, PRP was first incubated at 37 °C with BJ-PLA_2_-I (20.5 μg/mL) for 5 min, followed by addition of ADP (5 μM) and evaluation for another 10 min to determine the inhibition promoted by the toxin. Results were expressed as mean percentages ± SD (*n* = 2) of two independent experiments. *p < 0.05 compared to the ADP group

BJ-PLA_2_-I high enzymatic activity was shown by its high phospholipase activity, with 2.5 μg inducing an effect higher than that of 15 μg of *B. jararaca* venom (Fig. 5). In addition, our results showed that BJ-PLA_2_-I was able to inhibit the ADP-induced platelet aggregation by about 50% (Fig. 6).

### Evaluation of inflammatory effects

**Fig. 7:**
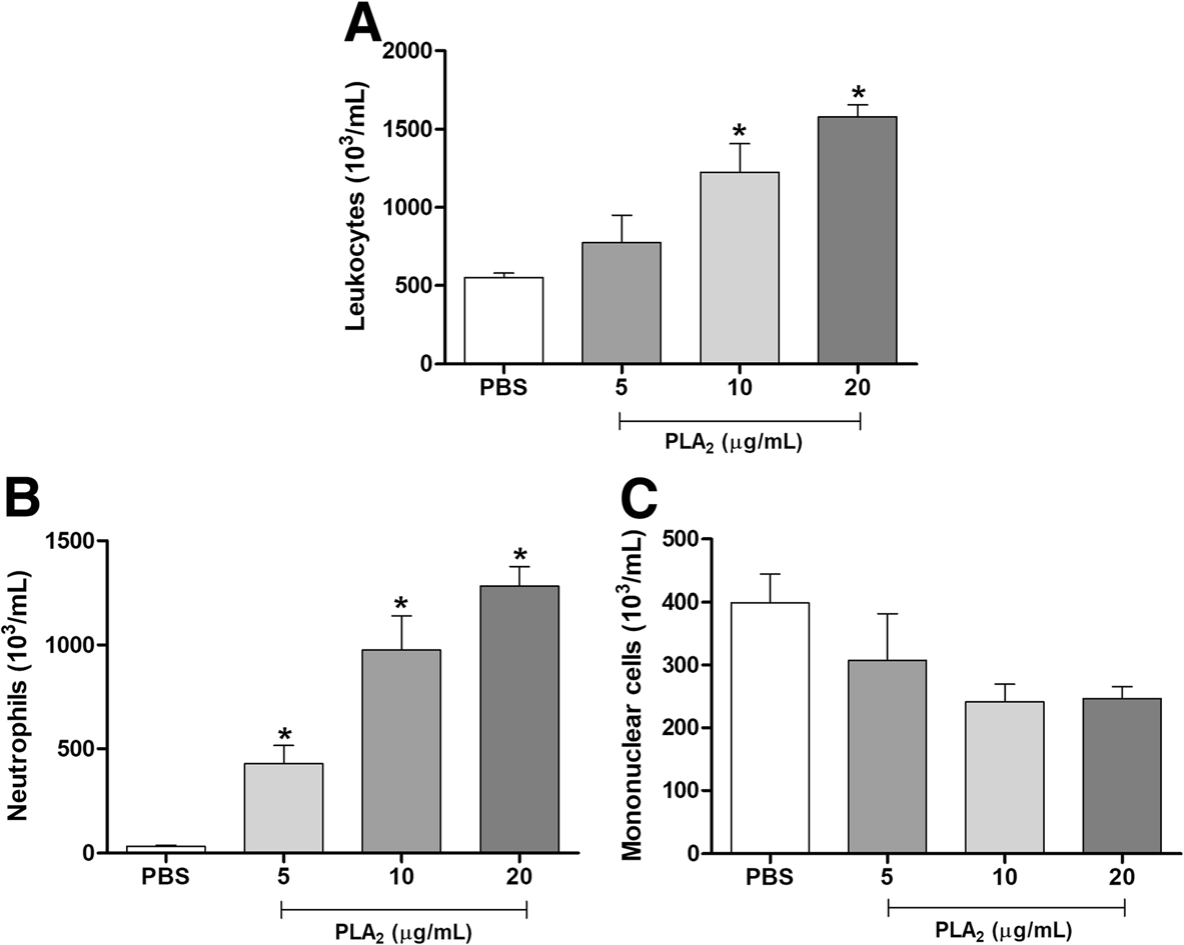
Leukocyte recruitment induced by different concentrations of BJ-PLA_2_-I in mice. Migration of total leukocytes (**a**), neutrophils (**b**) and mononuclear cells (**c**) was evaluated at 4 h after the injection of BJ-PLA_2_-I (5, 10 or 20 μg/mL) in the peritoneal cavity of mice. Control groups were injected only with PBS. Results were expressed as means ± SD (*n* = 5) of two independent experiments. *p < 0.05 compared to the control group (PBS)

The inflammatory effects of different concentrations of BJ-PLA_2_-I were initially evaluated by the influx of leukocytes into the peritoneal cavity of mice at 4 h after injection (Fig. 7). The toxin at 10 and 20 μg/mL increased the total number of leukocytes (Fig. 7a), while the number of neutrophils was increased at all concentrations evaluated (Fig. 7b) and the number of mononuclear cells did not change significantly in comparison to the PBS control (Fig. 7c).

**Fig. 8:**
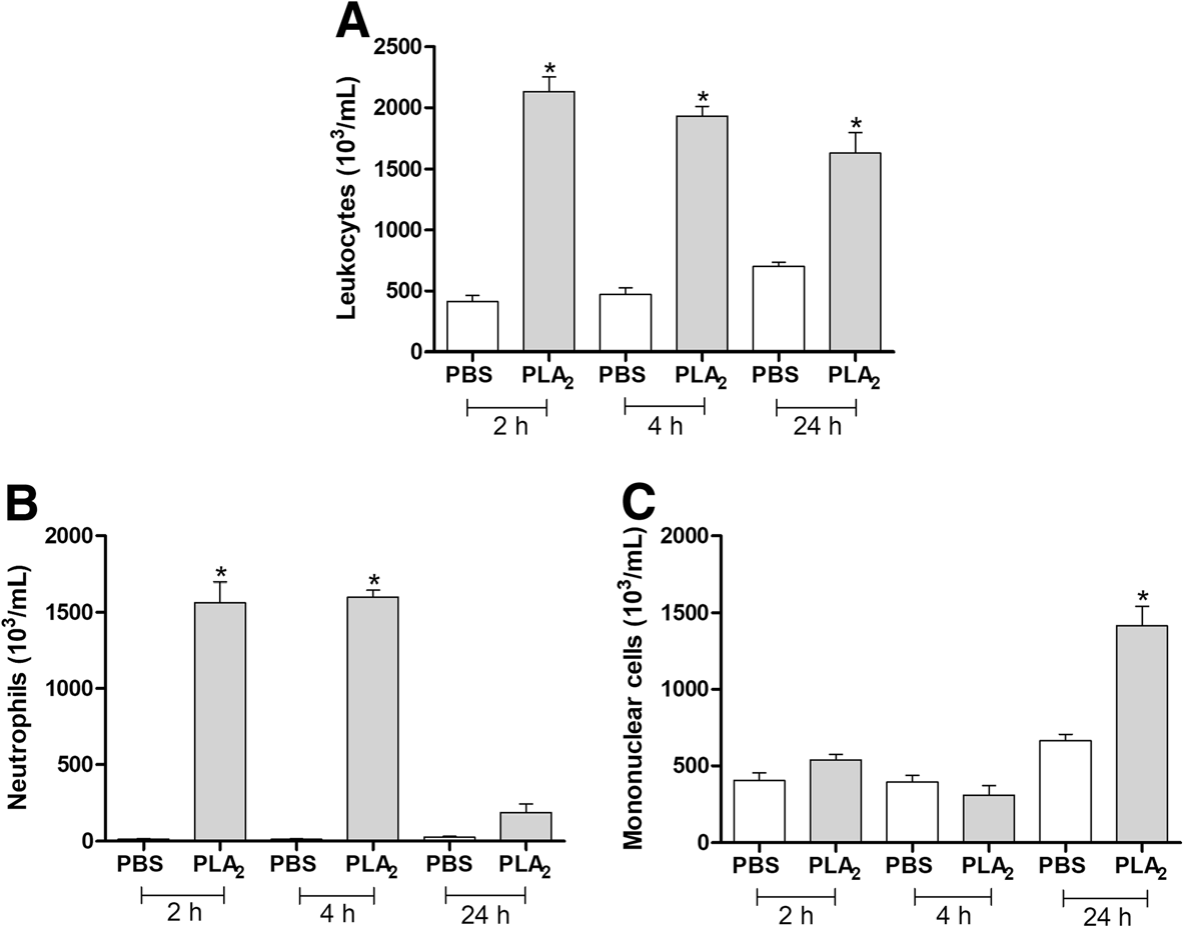
Leukocyte recruitment induced by BJ-PLA_2_-I in mice after different stimulation periods. Migrations of total leukocytes (**a**), neutrophils (**b**) and mononuclear cells (**c**) were evaluated at 2, 4 and 24 h after the injection of BJ-PLA_2_-I (10 μg/mL) in the peritoneal cavity of mice. Control groups were injected only with PBS. Results were expressed as means ± SD (*n* = 5) of two independent experiments. *p < 0.05 compared to the control group (PBS)

Afterward, a single concentration of BJ-PLA_2_-I (10 μg/mL) was employed to evaluate the leukocyte migration response at different periods (2, 4 and 24 h) after toxin injection (Fig. 8). Our results showed that this PLA_2_ induced increased leukocyte recruitment after all three stimulation periods evaluated (Fig. 8a), with significant increases of neutrophils at 2 and 4 h (Fig. 8b) and of mononuclear cells at 24 h (Fig. 8c).

**Fig. 9:**
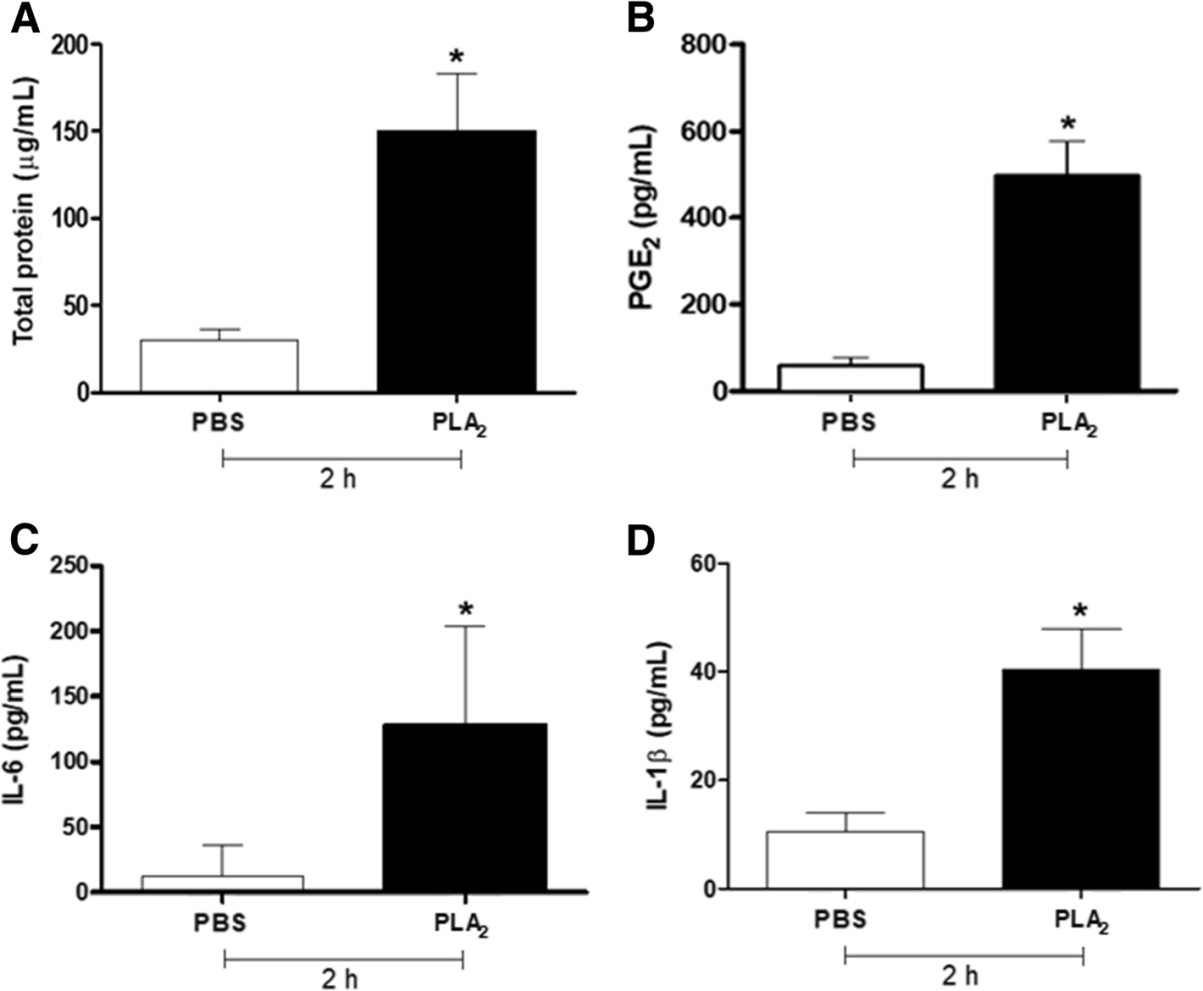
Increased levels of total proteins and inflammatory mediators induced by BJ-PLA_2_-I in mice. The cell-free supernatants from the peritoneal exudate of mice stimulated for 2 h with BJ-PLA_2_-I (10 μg/mL) were used for the quantification of total proteins (**a**), PGE_2_ (**b**), IL-6 (**c**) and IL-1β (**d**). Results were expressed as means ± SD (*n* = 5) of two independent experiments. *p < 0.05 compared to the control group (PBS)

Considering these results, we then investigated the protein extravasation and the production of inflammatory mediators induced by BJ-PLA_2_-I at 10 μg/mL after 2, 4 and 24 h. Compared to the PBS control, mice stimulated with BJ-PLA_2_-I did not show significant changes in the levels of mediators such as LTB_4_, TNF-α, IL-10 and NO (data not shown), but they did present increased levels of total proteins, PGE_2_, IL-6 and IL-1β only at 2 h after injection (Fig. 9).

### Cytotoxic effects

**Fig. 10:**
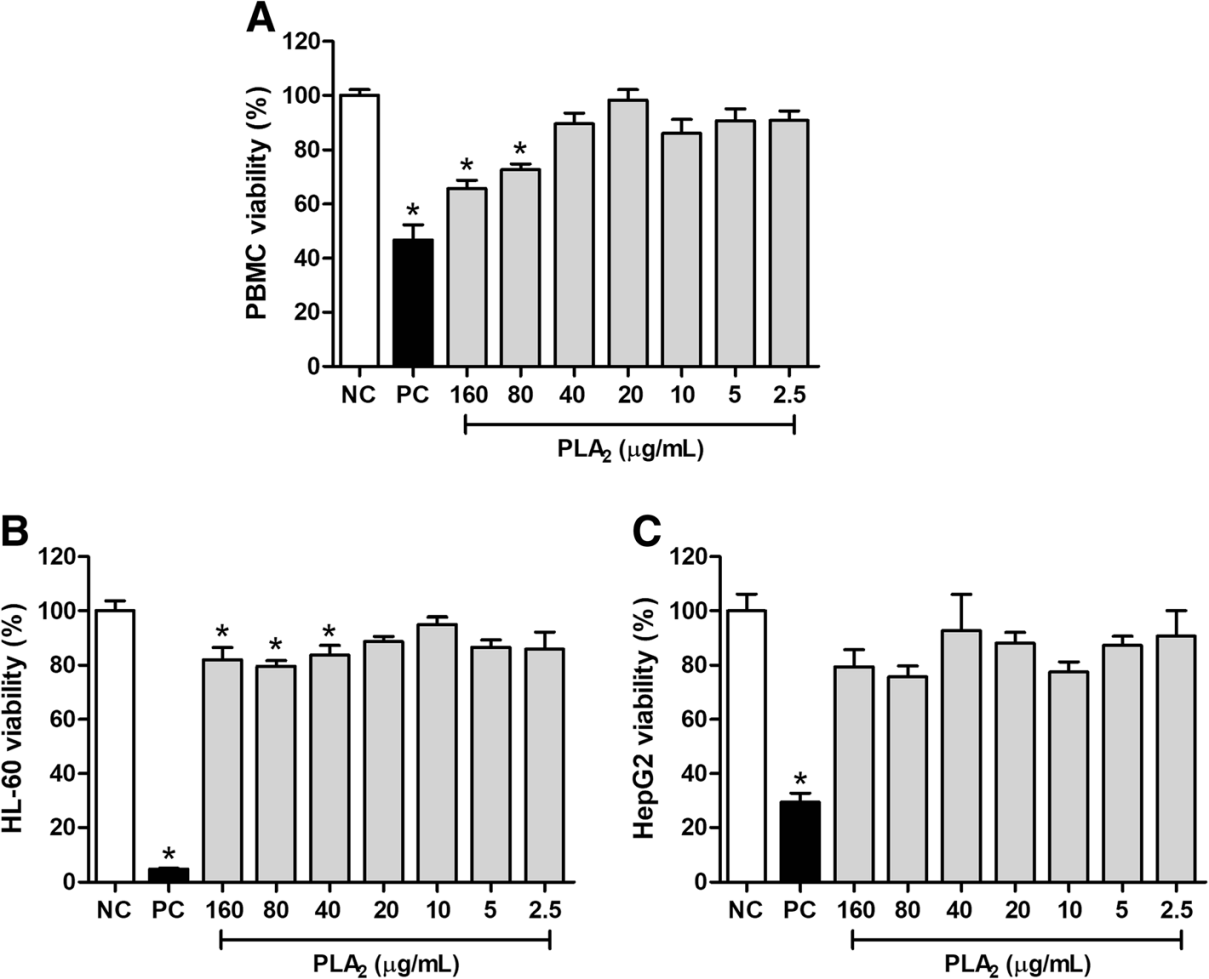
Evaluation of the cytotoxic effects induced by BJ-PLA_2_-I on normal and tumor cells. Cell viability of PBMC (**a**), HL-60 (**b**) and HepG2 (**c**) was assessed by the MTT method after a 24 h stimulation period with BJ-PLA_2_-I at different concentrations (2.5–160 μg/mL). Cells treated only with sterile PBS were used as negative controls (NC). The positive controls (PC) received cisplatin (Incel, Darrow®) at the final concentration of 250 μg/mL. Results were expressed as mean percentages (in relation to the negative control, considered as 100%) ± SD (*n* = 3) of two independent experiments. *p < 0.05 compared to the negative control (NC)

The cytotoxic effects of BJ-PLA_2_-I were assessed by treating normal cells (PBMC) or tumor cell lines (HL-60 and HepG2) with the toxin at different concentrations (2.5–160 μg/mL), followed by the determination of their cell viability. The results showed that BJ-PLA_2_-I was cytotoxic to PBMC at the two highest concentrations evaluated (80 and 160 μg/mL), as shown by the significant reduction in the PBMC viability in comparison to the negative control (Fig. 10a).

Regarding the tumor cell lines, BJ-PLA_2_-I significantly reduced the viability of HL-60 cells at the three highest concentrations assayed (40, 80 and 160 μg/mL) (Fig. 10b). HepG2 cell viability, on the other hand, was not altered by treatment with any of the BJ-PLA_2_-I concentrations evaluated (Fig. 10c).

## Discussion

Our study described the isolation and characterization of an acidic PLA_2_ from *B. jararaca* venom, which we named BJ-PLA_2_-I. Comparing our results with those described by Serrano et al. [28] for BJ-PLA_2_, an acidic PLA_2_ also from *B. jararaca* venom, we have strong evidence to indicate that both are the same toxin: molecular mass of 14,276 Da for BJ-PLA_2_-I vs. 14,289 Da for BJ-PLA_2_, besides 100% identity in the 60 first amino acid residues from their N-terminal and inhibition of the ADP-induced platelet aggregation with IC_50_ ~ 20.5 μg/mL [28]. However, as we did not determine the full amino acid sequence of our toxin, we chose to name it differently, specifically BJ-PLA_2_-I.

Although we used different purification procedures to obtain BJ-PLA_2_-I in comparison to Serrano et al. [28], the final yield for its purification was also very low (0.2% for BJ-PLA_2_-I vs. 0.35% for BJ-PLA_2_), which indicates that this toxin represents a very small fraction of the total protein content of *B. jararaca* venom. This is consistent with previous proteomic data on this venom, showing that PLA_2_s only represent about 3% of its protein content, a very low percentage when compared to other *Bothrops* venoms, such as that of *B. jararacussu* (~ 20% of PLA_2_s) [29].

BJ-PLA_2_-I showed high catalytic activity as evaluated by the phospholipase assays, which is consistent with the presence of the Asp49 residue and its classification as an acidic PLA_2_. Several other acidic PLA_2_s from *Bothrops* venoms have been described as exerting high catalytic activity, including Bl-PLA_2_ (*B. leucurus*) [30], Bp-PLA_2_ (*B. pauloensis*) [31], BpirPLA_2_-I (*B. pirajai*) [32], BmooPLA_2_ (*B. moojeni*) [33], BE-I-PLA_2_ (*B. erithromelas*) [34], MTX-I (*B. brazili*) [35] and BatroxPLA_2_ (*B. atrox*) [22].

Another BJ-PLA_2_-I feature we observed was its ability to inhibit the ADP-induced platelet aggregation. Numerous snake venom PLA_2_s have been reported to act on platelet functions, which allowed their classification into 3 groups: class A includes the PLA_2_s able to induce platelet aggregation; class B, PLA_2_s that inhibit platelet aggregation induced by several agonists; and class C, PLA_2_s that present biphasic responses in platelets (pro- and anti-aggregating properties) [36]. According to our results, BJ-PLA_2_-I can be classified into class B, along with other PLA_2_s such as BpirPLA_2_-I (*B. pirajai*) [32], BthA-I-PLA_2_ (*B. jararacussu*) [37], BE-I-PLA_2_ (*B. erythromelas*) [34], BpPLA_2_-TXI (*B. pauloensis*) [31] and BmooPLA_2_ (*B. moojeni*) [33].

Once purified and characterized, BJ-PLA_2_-I was assessed as to its inflammatory effects. This evaluation is important since inflammation is a typical process in envenomations by the Viperidae and Crotalidae snake families, whose effects triggered by the inflammatory reactions have not been properly neutralized by the usual anti-ophidian serum therapy [30, 38–40]. Furthermore, PLA_2_s are described as one of the major toxin classes responsible for the inflammatory effects induced after snake envenomations [41].

The inflammatory potential of BJ-PLA_2_-I was initially assessed by in vivo leukocyte infiltration experiments. Leukocyte migration is a process involving several steps that are mediated by a dynamic of interaction between adhesion molecules expressed by leukocytes and endothelial cells, an expression that is regulated by cytokines and chemokines [42].

In general, administration of BJ-PLA_2_-I induced pronounced leukocyte infiltration in the peritoneal cavity of mice, formed mainly by neutrophils in the first hours (2 and 4 h) and by mononuclear cells after 24 h. These effects are not surprising since several studies have already shown that toxins from different classes (including PLA_2_s, serine and metalloproteases, L-amino acid oxidases and cysteine-rich secretory proteins) can promote inflammatory responses related to the infiltration of leukocytes [24, 43–46]. Interestingly, some studies have shown that catalytically inactive PLA_2_s (Lys49 PLA_2_s) can also induce leukocyte migration similar to those of catalytically active enzymes (Asp49 PLA_2_s), which suggests that the catalytic activity is not strictly necessary to trigger inflammatory responses, although it may contribute to these effects [6, 41, 47]. This is reinforced by studies using PLA_2_s chemically modified by BPB (p-bromophenacyl bromide, a classic PLA_2_ inhibitor), which demonstrated that these molecules did not lose their inflammatory effects [6, 48].

Besides inducing leukocyte infiltration, BJ-PLA_2_-I was also involved in the increased production of inflammatory mediators, including some cytokines (IL-6 and IL-1β) and eicosanoids (PGE_2_), and increased levels of total proteins in the peritoneum of mice, which indicate extravasation of proteins due to possible edematogenic effects of the toxin. On the other hand, levels of LTB_4_, IL-10, TNF-α and nitric oxide were not altered after stimulation with BJ-PLA_2_-I. Taking all these findings into account, the results for BJ-PLA_2_-I indicate a local inflammatory response, similar to the ones previously described for other Asp49 PLA_2_s from *Bothrops* venoms, such as BatroxPLA_2_ (*B. atrox*) [24], MT-III (*B. asper*) [6]; Bl-PLA_2_ (*B. leucurus*) [30] and Bleu-TX-III (*B. leucurus*) [49].

Activated leukocytes release a broad spectrum of cytokines, as well as proteins that contribute to the inflammatory process. The cytokines IL-6, IL-10, IL-1β and TNF-α are the main regulators of the inflammatory response, being able to induce fever, expression of adhesion molecules and activation of T and B cells [50]. Inflammatory events can also be attributed to the release of lipid mediators, including prostaglandins, thromboxanes and leukotrienes [12, 51]. PGE_2_ is an important member of the prostaglandin family that plays several roles in inflammation, exerts immunomodulatory effects, acts as a potent vasodilator and induces bradykinins. PGE_2_ is also known to suppress production of TNF-α, in addition to inhibiting T cell proliferation [52, 53]. Considering that TNF-α induces the synthesis of substances that cause tissue damage, such as nitric oxide [54], the increased levels of PGE_2_ induced by BJ-PLA_2_-I could be related to the unaltered levels of TNF-α and nitric oxide. In addition, production of PGE_2_ but not of LTB_4_ might indicate that BJ-PLA_2_-I-induced inflammation is related to the cyclooxygenase (COX) pathway instead of the lipoxygenase (LOX) one.

In our study, we showed that, overall, BJ-PLA_2_-I presented low cytotoxic effects on normal (PBMC) and tumor cells (HL-60 and HepG2), with viabilities mostly varying between 70 and 80% even at the highest concentrations. Such low cytotoxic effects have been attributed to other acidic PLA_2_s as well. De Albuquerque Modesto et al. [34] evaluated the cytotoxic potential of BE-I-PLA_2_, an acidic PLA_2_ from *B. erithromelas* venom, in human umbilical vein cells (HUVEC), showing that this PLA_2_ was not toxic to these normal human cells. Similar effects were described by Nunes et al. [30] for Bl-PLA_2_, an acidic PLA_2_ from the *B. leucurus* venom, which displayed low cytotoxicity to PBMC. On the other hand, there are also reports of acidic PLA_2_s with significant cytotoxic effects on different tumor cell lines. Roberto et al. [37] assessed the cytotoxic potential of BthA-I-PLA_2_ from *B. jararacussu* venom against three tumor cell lines: Jurkat (leukemic cells), SK-BR-3 (human breast tumor cells) and EAT (Ehrlich ascites tumor cells). BthA-I-PLA_2_ at 100 μg/mL was demonstrated to be highly cytotoxic to Jurkat and SK-BR-3 (50 and 30% viability, respectively), while the viability of EAT cells was less affected (80% viability). Likewise, the acidic PLA_2_s BmooTX-I from *B. moojeni* venom [33] and MTX-I from *B. brazili* venom [35], at a concentration of 100 μg/mL, reduced the viability of Jurkat cells to 50 and 40%, respectively.

Despite inducing low cytotoxicity in the tumor cells evaluated, we observed that BJ-PLA_2_-I significantly reduced the viability of HL-60 cells, but not that of HepG2 cells. This different cytotoxic specificity may be related to several factors, including the fact that HL-60 cells grow as a suspension, while HepG2 are adherent cells. Nevertheless, the opposite behavior was described for nigexine, a PLA_2_ from *Naja nigricollis* venom, which was more cytotoxic to adherent cell lines (epithelial FL and C-13 T neuroblastoma cells) than to those in suspension (HL-60) [55].

Thus, although some snake venom PLA_2_s can present cytotoxic effects, most of these enzymes do not exhibit this activity, which strongly suggests that other mechanisms, unrelated to the PLA_2_ catalytic activity, are involved in the cytotoxicity [55, 56]. In fact, Lomonte et al. [57] identified a region near the C-terminal of Lys49 PLA_2_ homologues responsible for their cytotoxic effects. This would explain why some Lys49 PLA_2_s, which typically lack catalytic activity, are also described as cytotoxic molecules [7, 56].

## Conclusions

BJ-PLA_2_-I was successfully isolated from *B. jararaca* venom and characterized as an acidic Asp49 PLA_2_ that induces acute local inflammation in mice and low cytotoxicity in normal (PBMC) and tumor cells (HL-60 and HepG2). The information obtained in the present work brings significant contributions to the studies of animal toxins, both in relation to *Bothrops* envenomations and the understanding of the mechanisms involved in the biological effects induced by PLA_2_s. Thus, BJ-PLA_2_-I may contribute to the biotechnology field, by serving as a molecular model for the formulation of more effective drugs used in the treatment of various diseases or even for developing novel strategies for anti-ophidian therapy.
